# Choline: An Essential Nutrient for Skeletal Muscle

**DOI:** 10.3390/nu12072144

**Published:** 2020-07-18

**Authors:** Antimo Moretti, Marco Paoletta, Sara Liguori, Matteo Bertone, Giuseppe Toro, Giovanni Iolascon

**Affiliations:** Department of Medical and Surgical Specialties and Dentistry, University of Campania “Luigi Vanvitelli”, 80138 Naples, Italy; marco.paoletta@unicampania.it (M.P.); sara.liguori@unicampania.it (S.L.); bertonematteodott@gmail.com (M.B.); giuseppe.toro@unicampania.it (G.P.); giovanni.iolascon@gmail.com (G.I.)

**Keywords:** choline, skeletal muscle, striated muscle, review, muscle fat, muscle protein, autophagy, inflammation, muscle performance, vitamin B complex

## Abstract

Background: Choline is an essential micronutrient with a pivotal role in several metabolic pathways contributing to liver, neurological, and hematological homeostasis. Although choline is commonly administered to improve physical performance, its effects on muscle are still unclear. The aim of this scoping review is to analyze the role of choline on skeletal muscle in terms of biological effects and clinical implications. Methods: A technical expert panel (TEP) of 6 medical specialists with expertise in muscle physiology and skeletal muscle disorders performed the review following the PRISMA-ScR (Preferred Reporting Items for Systematic Reviews and Meta-Analyses Extension for Scoping Reviews) model. The TEP planned a research on PubMed selecting “choline” as MeSH (Medical Subject Headings) term adding to PubMed Search Builder the terms ”skeletal muscle” and “muscle striated”. TEP considered for eligibility articles published in the last 30 years, including original researches, particularly in vitro studies, and animal and clinical studies in the English language. Results: From the 1239 studies identified, TEP included 14 studies, 3 in vitro, 9 animal, and 2 clinical studies. Conclusions: Our scoping review elucidates and summarizes the crucial role of choline in modulating muscle fat metabolism, muscle proteins homeostasis, and the modulation of inflammation and autophagy.

## 1. Introduction

Choline is a water-soluble quaternary amine of the vitamin B group considered as an essential nutrient by the Food and Nutrition Board of the Institute of Medicine [1]. Choline endogenous synthesis from amino acid methionine is insufficient to support human choline requirements, so it is essential to maintain an appropriate dietary intake of choline, consuming fish, eggs, and meats [2]. Adequate choline daily intakes ranged from 330 to 468 mg for men, 269 to 444 mg for women, and 356 mg/day as mean estimate intake in pregnant women [3]. This micronutrient plays an important role in phospholipid synthesis and triglycerides metabolism contributing to structure and function of cell membranes, including skeletal muscle cells [4]. Low concentration of choline is associated with several changes in myoblasts up to muscle wasting as demonstrated by increased serum creatine kinase (CK) [5]. Moreover, choline seems to exert an ancillary role in inflammatory muscle diseases with anti-fibrotic effects [6]. From a functional point of view, choline is involved in muscle contraction being a precursor of the main neurotransmitter of α-motor neurons, acetylcholine (ACh) [7]. These effects might have clinical implications as suggested by the observation of poor physical performance in healthy people with low serum choline [8,9,10].

Despite available knowledge about effects of choline on both muscle histomorphology and function, pathways involved in these processes are still poorly investigated.

The aim of this scoping review is to analyze the state of the art regarding the role of choline on skeletal muscle in terms of biological effects and clinical implications.

## 2. Materials and Methods

This scoping review was performed following the PRISMA-ScR (Preferred Reporting Items for Systematic Reviews and Meta-Analyses Extension for Scoping Reviews) model [11]. First step was the creation of a technical expert panel (TEP) consisting of 6 medical specialists with expertise in muscle physiology and skeletal muscle disorders. All TEP components are confident with scoping review methodology. As search strategy, the TEP planned a research on PubMed (Public MedLine, run by the National Center of Biotechnology Information, NCBI, of the National Library of Medicine of Bethesda, Bethesda, MD, USA), selecting “choline” as MeSH (Medical Subject Headings) term; the following terms were added to run the PubMed Search Builder: ”skeletal muscle”, “muscle striated” (“Choline” (Mesh) AND “skeletal muscle” (Mesh)) and (“Choline” (Mesh) AND “muscle striated” (Mesh)).

According to the aim of the review, the TEP defined the characteristics of the sources of evidence, considering for eligibility any researches published in scientific literature in the last 30 years (last update on 30th April 2020), including original articles, particularly in vitro studies, and animal and clinical studies in English language.

## 3. Results

**Figure 1:**
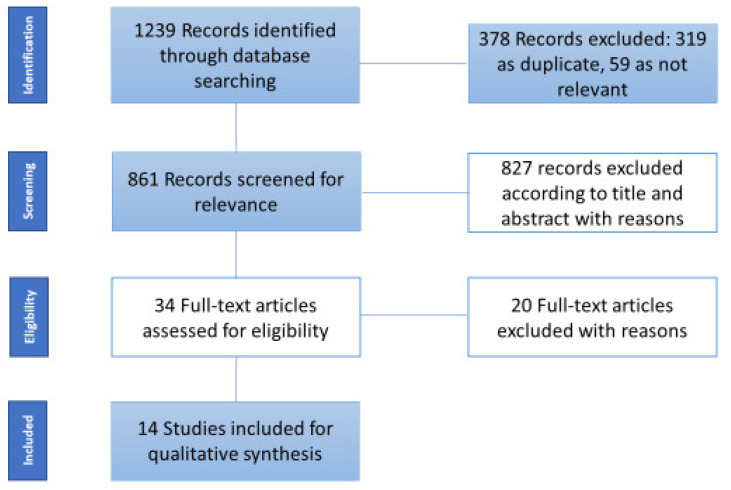
Flow diagram of sources selection process.

From the 1239 studies identified, 861 articles were screened for eligibility after removing duplicates (*n* = 319) and 59 articles were not relevant to the objective of the scoping review. Of these, 827 were excluded for different reasons (471 considered choline exclusively as neurotransmitter (i.e., acetylcholine, succinylcholine, and carbacholine), 275 were referred exclusively to smooth or cardiac muscle, 59 provided choline as component of platelet-activating factor, and 22 considered choline as fluoromethylcholine or other radioactive tracers). Thirty-four articles were examined in full text. Of these, only 14 studies were included (20 articles were excluded for the following reasons: 11 were referred to choline as neurotransmitter, 6 were not relevant for scoping review purpose, 1 was not an original article, 1 was about radioactive tracer, and 1 was not written in English language) (Figure 1). We divided our results in preclinical and clinical studies (Table 1).

### 3.1. Pre-Clinical Studies

In our scoping review, we included twelve preclinical studies consisting of three in vitro and nine animal model studies.

#### 3.1.1. In Vitro Studies

Beca et al. [12] explored the effect of monovalent cations including choline^+^ on Ca^2+^ transport through sarcoplasmic/endoplasmic reticulum Ca^2+^ ATPase (SERCA) activity. SERCA pumps are responsible for Ca^2+^ ions accumulation in sarcoplasmatic reticulum (SR) against a concentration gradient. The action of these pumps is increased by the presence of monovalent cations such as K^+^. The study demonstrated that the replacement of K^+^ with choline^+^ had potent inhibitory effects on SERCA and Vmax of Ca^2+^ uptake into the SR in rabbit skeletal muscle.

Kovacs et al. [13] examined the role of sphingosylphosphorylcholine (SPC) in the regulation of ryanodine receptors (RyRs) in SR vesicle preparations. RyRs are channel complexes expressed on endoplasmic reticulum of skeletal muscle (RyR1), cardiac muscle (RyR2) and other tissues (RyR3). RyR1 mobilizes intracellular Ca^2+^ depending by cytosolic Ca^2+^ concentration and Ca^2+^ sensor calmodulin (CaM) that have inhibitory effects on channel activity. Below the critical micellar concentration (CMC), SPC directly inhibited RyR1 and, above CMC, SPC displaced Ca^2+^ CaM from the RyR1.

Michel et al. [14] investigated the effects of choline deficiency in C2C12 muscle cells observing a 40% reduction in mitochondrial choline uptake and a down-regulation of choline-specific transporter SLC44A1 mRNA level; moreover, choline deficiency reduced phosphatidylcholine (PC) synthesis and TAG degradation in muscle cells increasing TAG accumulation.

#### 3.1.2. Animal Studies

Kenney et al. [15] evaluated the effects of 0.18% choline and 0.5% myo-inositol supplementation versus chow only on levels of liver and carcass fat in non-deficient aerobically trained rats. Authors hypothesized that these supplements enhance the burning of stored fat and are used by athletes to improve physical performance by increasing muscle-to-fat ratio. This study demonstrated that diet supplemented with choline and myo-inositol decreased the percentage of fat within liver and weight gain compared to control group. However, no between-group difference was found in term of carcass fat, suggesting no effect of these substances on reducing fat mass.

Eder [16] investigated if hyperlipidemic diet (cholesterol and cholic acid) associated to low choline concentrations produced a lipid accumulation in liver and muscle tissue in geese; moreover, in this study diet was supplemented with methionine, a methyl donor useful to synthetize choline, at lower level recommended. Choline, as PC precursor, is implicated in the secretion of lipids from liver into plasma as very low-density lipoproteins, and its deficiency results in lipid accumulation and reduced PC levels in both liver and plasma. However, authors observed that PC concentrations were not influenced by low dose of choline or by cholesterol and cholic acid supplementation in both hepatic and breast muscle tissues. These findings suggested that there was sufficient endogenous choline synthesis, probably from methionine, in this animal model.

Ilcol et al. [17] demonstrated that intravenous endotoxin infusion modifies serum-free and serum phospholipid-bound choline concentrations in a dose-related manner. Low dose of endotoxin (0.02 mg/kg) reduced serum free choline and increased serum phospholipid-bound choline. Administration of 1 mg/kg of endotoxin resulted in higher levels of both serum free and phospholipid-bound choline, as a consequence of enhanced choline release by damaged tissue along with a poor choline clearance due to liver and kidney failure; however, serum choline increase might be confounded by endotoxin-induced dehydration. Finally, intravenous administration of choline (20 mg/kg) 5 min before, and up to 8 h after administration of 1 mg/kg of endotoxin demonstrated to hamper endotoxin-induced tissue injury, also at skeletal muscle level; this protective role might be explained by enhanced cholinergic neurotransmission thanks to increased free choline availability, improved tissue perfusion, and reduced endotoxin-related hypotension.

Schenkel et al. [18] investigated the effects of different amounts of two fatty acids (FAs), namely palmitic acid (PAM) and oleic acid (OLA), on expression and content of choline transporter-like protein 1 (CTL1/SLC44A1) in C2C12 mouse myoblasts. CTL1/SLC44A1 is a plasma membrane transporter that regulates intracellular choline requirements in skeletal muscle by modulating PC synthesis through the Kennedy or cytidine triphosphate (CDP)-choline pathway. It seems that choline deficiency downregulates this transporter thus redirecting Kennedy pathway to TAG synthesis and lipid droplets accumulation. Both PAM and OLA did not decrease CTL1/SLC44A1 mRNA expression while PAM reduced plasma membrane CTL1/SLC44A1 content as a consequence of increased protein degradation; on the other hand, OLA reduced mitochondrial membrane CTL1/SLC44A1 content. PAM, but not OLA, reduced choline uptake. Both these FAs reduced mitochondrial choline uptake, mitochondrial function, as well as the membrane potential. Finally, OLA increased de novo PC synthesis in muscle cells avoiding the accumulation of toxic lipid intermediates, such as DAG.

Li et al. [19] demonstrated that diet supplementation of 0.25% rumen-protected choline (RPC) increased growth and intramuscular fat compared to controls and to higher RCP regimens (0.50% and 0.75%) in lambs. Moreover 0.25% RPC increased the expression of genes regulating lipogenesis, particularly of those involved in intramuscular fat deposition, including cluster of differentiation 36 (CD36), acetyl-CoA carboxylase (ACC), and fatty-acid synthase (FASN) compared to other groups, whereas supplementation of 0.75% RPC increased lipoprotein lipase (LPL) and FASN genes and decreased ACC gene without any significant effects on CD36 gene expression.

Oster et al. [20] examined the effects of a methylating micronutrient-rich maternal diet (MET), compared to standard diet on fetuses of pietrain gilts at three prenatal time points (35, 63 and 91-days post-conception, dpc) on fetal muscle and liver tissue. MET and standard diet included different levels of methionine (4700 mg/kg versus 2050 mg/kg), choline (2230 mg/kg versus 500 mg/kg), folic acid (92.2 mg/kg versus 3 mg/kg), vitamin B6 (1180 mg/kg versus 3 mg/kg), vitamin B12 (5930 μg/kg versus 31 μg/kg), and zinc (149 mg/kg versus 21.8 mg/kg). Authors demonstrated that fetuses of gilts fed with MET showed an increased fetal weight compared to those fed with standard diet, in particular at 35 and 91 dpc time points. At muscle tissue, diet alone did not modify levels of insulin-like growth factor (IGF) and IGF binding proteins (IGFBPs). However, an interaction between diet and stage of development modulated the expression of IGF signaling on fetuses’ muscle (M. longissimus dorsi) increasing IGF2 level and reducing insulin-like growth factor binding proteins (IGFBPs) in MET muscle.

Robinson et al. [21] demonstrated that dietary methyl donors including choline contribute to respond to methionine requirement and protein homeostasis in neonatal piglets. Methyl-deficient (MD) diet results in reduced whole body and synthesis, breakdown, and metabolism of muscle proteins compared to methyl-sufficient (MS) diet (60 mg choline/ (kg × day)).

Taylor et al. [22], analyzed effects of choline supplementation (2 mg/mL choline in drinking water for 4 weeks, ~240 μg/g/day) versus no intervention on muscle function, in insulin resistant (IR) 21 CTP:phosphoethanolamine cytidylyltransferase deficient (Pcyt2^+/−^) mice. Authors demonstrated that choline increased content of membrane phospholipids (PC and sphingomyelin), DAG, and glycogen levels (+30% and +60%, respectively). On the other hand, choline intake reduced both TAG and FA muscle content (−40% and −60%, respectively), compared to wildtype littermate control (Pcyt2+/+). Furthermore, the same intervention reduced de novo FA synthesis and lipogenesis, improved mitochondrial energy production restoring the activity of metabolic regulators like AMP-activated protein kinase (AMPK), and increased muscle FA oxidation up to controls level.

Jahanian et al. [23] compared the effects of diets at different metabolizable energy (ME) levels (basal, +0.42 and +0.84 MJ/kg ME) with supplementation of choline and carnitine on both growth and structural characteristics of skeletal muscle in chicken at starter, grower and finisher periods (1 to 14 days of age, 15 to 28 days of age and 29 to 42 days of age, respectively). Authors found that 0.42 MJ/kg ME combined with choline increased average daily feed intake (ADFI) during the grower period and decreased ADFI during the starter and finisher periods. Moreover, both choline and carnitine supplementation (1000 mg/kg and 100 mg/kg, respectively), increased moisture and reduced malondialdehyde (MDA) content in leg muscle, whereas choline supplementation alone reduced MDA and increased protein content in breast muscle.

### 3.2. Clinical Studies

Fisher et al. [24] conducted a study on 57 participants received a diet containing 550 mg choline·70 kg body wt^−1^·day^−1^ and 50 mg betaine·70 kg body wt^−1^·day^−1^, and 400 dietary folate equivalents (DFE)/day (baseline) followed by a choline-depletion stage (< 50 mg choline·70 kg body wt^−1^·day^−1^ and 6 mg betaine·70 kg body wt^−1^·day^−1^). This depletion phase continued for 42 days or until the subjects developed an organ dysfunction defined as 5-fold increase in serum creatine phosphokinase (CPK) activity, a 1.5 fold increase in aspartate aminotransferase (AST), alanine aminotransferase (ALT), γ- glutamyltransferase (GGT), or lactate dehydrogenase (LDH), or a liver fat content higher than 28%. Thirty-nine subjects became choline-deficient, of which 6 (early-depleters) during the 550-mg choline diet, and 33 during the low choline diet. In five of the six early depleters muscle involvement was documented by increased serum CPK (> 7000% compared to baseline value). Similarly, of the subjects in low choline-regimen, only one subject experimented a muscle involvement, whereas six had a combination of muscle and liver impairment. The remaining 26 subjects experienced only signs of liver dysfunction. In subjects on low choline-regimen with organ involvement mean serum CPK was 803 U/L, and for the subgroup of seven subjects with muscle dysfunction (six muscle plus liver, and one muscle only), the mean CPK value was 3993 U/L (maximum value observed 6849 U/L). Despite muscle damage, no electrodiagnostic sign or muscle symptoms (e.g., pain) were reported and complete recovery after choline repletion was observed.

Mc Lean et al. [25] measured 2000-m time-trial running performance, serum hemoglobin, and intramuscular carnosine (a muscular buffer) in a group of athletes after a 19-day of training performed at moderate altitude (ALT) or at sea level (CON). Authors demonstrated that athletes improved performance after ALT (+1.5%) compared to CON and these changes persisted after four weeks from end of training period. In the ALT group, serum hemoglobin increased by 3.6% and returned to the baseline values after 30 days. It should be underlined that the authors did not find any change in intramuscular levels of carnosine both in gastrocnemius and soleus after ALT, and an increase in the carnitine/choline peak (8.8% ± 6.1%) was observed only in soleus.

## 4. Discussion

To the best of our knowledge, this is the first scoping review that has investigated the effects of choline on skeletal muscle. Choline is an essential nutrient with many biological implications as a structural component of cells membrane, as a precursor of cholinergic neurotransmitters and as a main actor of methylation pathways [26]. Therefore, this micronutrient has a pivotal role in liver, neurological, and hematological functions [27,28,29]. However, the effects of choline on skeletal muscle are still not well known. According to available literature choline affects skeletal muscle by modulating fat and protein metabolism, inflammation, and autophagy.

### 4.1. Choline and Muscle Fat Metabolism

Adequate amount of choline improves mitochondrial energy metabolism and lipid metabolism by decreasing FA synthesis. Choline deficiency impairs the incorporation of FAs in PC, increasing their availability for DAG and TAG synthesis, and consequently favoring accumulation of TAG in muscle cells [14]. Choline influences fat metabolism also modulating expression of genes involved in FA genesis (ACC and FASN) as well as those involved in fat intracellular transportation (LPL and CD36) [19]. Moreover, high levels of choline reduce biosynthesis of long-chain FAs in muscle lowering intramuscular fat content. This effect could be explained also by an insulin-mediated gene modulation. In fact, choline supplementation improves insulin signaling that upregulates AMPK, downregulates mTORC1, a modulating factor of proteostasis in skeletal muscle [30], and decreasing lipogenesis [31].

Choline also downregulates Diacylglycerol O-acyltransferase (Dgat1/2), reducing FA esterification and TAG formation. On the other side, an experimental study reported that additional choline supplementation did not result in significant difference in “carcass fat levels” when compared to control [15]. Accumulation of lipid intermediates in skeletal muscle (intermuscular and/or intramuscular adipose tissue, IMAT) leads to cellular dysfunction and death. An excess of lipids in diet could impair choline metabolism. This effect reduces the consumption of lipids for intracellular synthesis of PC, increasing DAG and FA accumulation, causing adipocyte hyperplasia and hypertrophy, chronic inflammation, lipotoxicity, and insulin resistance, key mechanisms involved in sarcopenic obesity [32]. However, it should be underlined that polyunsaturated FAs, such as PAM, reduce the expression of plasma membrane choline transporter CTL1/SLC44A1 inducing cell membrane fragility because of poor choline availability for PC synthesis. Moreover, choline deficiency shifts CDP-choline pathway toward TAG formation and lipid droplets accumulation resulting in lipotoxicity. Conversely, monounsaturated FAs, such as OLA, seem to exert a protective activity on muscle by increasing mitochondrial FA oxidation and stimulating PC synthesis. In a clinical scenario, Gao et al. demonstrated that choline supplementation improved body composition by lowering body fat and increasing lean mass, suggesting a pivotal role of this micronutrient in promoting FA β-oxidation and translocation into the mitochondria [33].

### 4.2. Choline and Muscle Proteins

**Figure 2:**
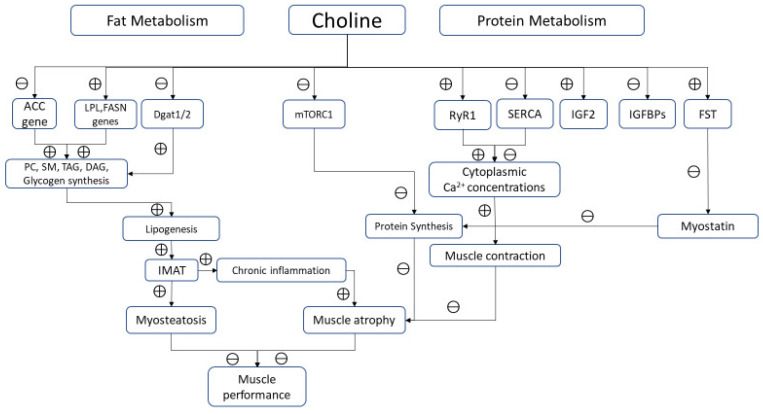
Biological pathways modulated by choline in skeletal muscle. Note: ⊕ and ⊖ indicate positive and negative modulation, respectively.

Choline is an essential nutrient for protein metabolism. As methyl-group donor, this micronutrient influences protein homeostasis, increasing synthesis and reducing breakdown. Robinson et al. reported a reduced whole-body protein synthesis in piglets fed with methyl deficient diet, due to 50% reduction of protein synthesis in skeletal muscle [21]. In the early stages, impaired protein synthesis did not lead to a poor body growth, probably because of a simultaneous slow protein catabolism. However, a chronic choline diet restriction undoubtedly determines a low muscle protein content, resulting in impaired muscle growth and function. Vice versa, choline-rich diet increases serum IGF2 and decreases IGFBP-2 in skeletal muscle fibers. IGF2 enhances proliferation and growth of muscle tissue promoting amino acid and glucose uptake [20,34]. It has been also hypothesized that methyl-donors nutrients, including choline, increases the expression of follistatin (FST), a member of the TGF-β family that causes hypertrophy and hyperplasia of muscle cells through binding ACTIIB receptor, and thus inhibiting myostatin [35,36] (Figure 2).

From a functional point of view, potential actions of choline in modulating key mechanisms of muscle contraction beyond its role as precursor of Ach should be considered. Ions replacement of K^+^ with choline+ results in a potent inhibition of SERCA in sarcoplasmic/endoplasmic reticulum of skeletal muscle as demonstrated in animal models [12]. This effect is reached through a dual mechanism: choline^+^ inhibits both the cytosolic Ca^2+^ uptake into SR and, at the same time, SERCA ATPase activity, probably uncoupling Ca^2+^ transport from ATP hydrolysis.

Choline regulates intracellular calcium and therefore muscle contraction also by modulating the binding of calmodulin and RYR1 [13]. This mechanism could increase the cytoplasmic calcium concentrations into myofibrillar spaces improving its bioavailability for muscle contraction. However, this seems not true in muscle affected by pathological conditions. Alves et al. reported that choline supplementation (5 g/kg choline) paradoxically increased SERCA activity reducing calcium cytosolic content in mice models of Duchenne Muscular Dystrophy (mdx) [6].

### 4.3. Choline and Inflammation

Choline influences inflammation through different mechanisms. In animal exposed to endotoxin choline improves activation of vagal anti-inflammatory system [17,37,38]. In the same animal model, choline counteracts the endotoxin-induced tissue damage reducing serum levels of urea, uric acid, lactate dehydrogenase (LDH), creatine kinase (CK), and creatine kinase myocardial isoform (CK-MB) [20], probably through improved tissue perfusion as well as enhanced cholinergic neurotransmission.

### 4.4. Choline, Apoptosis, and Autophagy

Choline modulates cell apoptosis and autophagy; thus, contributing to maintain intercellular homeostasis. Da Costa et al. [39] reported that choline deficiency was associated with significant DNA damage resulting in lymphocyte apoptosis. Authors found an increase of activated caspase-3 in lymphocytes in patients fed with choline deficient diet. This could be the main mechanism of apoptosis, probably occurring earlier than the reduced cytoplasmatic availability of choline for PC synthesis. Lower membrane PC concentration seems to contribute to plasma membrane fragility of myocytes [5].

Choline is an important regulator of autophagy. Taylor et al. reported that choline supplementation restores insulin receptor substrate 1 (IRS1) levels, a key factor in the IGF1-Akt-mTOR pathway with significant anabolic effects on skeletal muscle [31]. Choline prevents phosphorylation of mTORC1, defined as “the main gateway to autophagy” [40]. Autophagy is a critical survival mechanism of cells and its alteration conduce to skeletal muscle damage in different disorders. For example, Pompe disease, an inherited deficiency of the lysosomal enzyme acid α-glucosidase, is characterized by significant accumulation of autophagosomes containing LC3 (microtubule-associated protein 1 light chain 3), a pivotal factor for autophagy in type I muscle fibers. Two forms of LC3 have been described: a cytoplasmic protein (LC3-I), and a specifically associated autophagosomes form (LC3-II). Higher levels of LC3-II provoke a dramatic and disruptive autophagic effect on skeletal muscle [41]. Choline reduces lipidation of LC3, lowering LC3-II/LC3-I ratio and its accumulation in autophagy-lysosomal system [42]. This ratio is also influenced by FAs. PAM increases intracellular content of PC precursor providing more lipids for LC3 lipidation and autophagosome formation stimulating autophagy.

## 5. Conclusions

Our scoping review highlighted the effects of choline on skeletal muscle, describing its role in several pathways involved in fat and protein metabolism, inflammation, as well as autophagy. Adequate dietary intake of this micronutrient is required to properly modulate fat and protein metabolism decreasing FA synthesis and contributing to muscle growth and function, respectively. Moreover, choline promotes intercellular homeostasis counteracting inflammation, apoptosis, and autophagy. However, evidence supporting the benefits of choline on muscle derives mainly from basic research with few clinical studies. Therefore, further research looking at the clinical implications of choline supplementation on the structural characteristics and functional outcomes in human skeletal muscle is warmly required.

## Figures and Tables

**Table 1:** Relevant data from each study included in the scoping review.

Author, Year	Design	Main Results
Beca et al. 2009 [12]	In vitro	K^+^ replacement with Choline^+^ had inhibitory effects on the Vmax of Ca^2+^ uptake and, directly, on SERCA activity of SR in both canine cardiac and rabbit skeletal muscle.
Kovacs et al. 2010 [13]	In vitro	SPC levels below CMC directly inhibited skeletal muscle RyR1. SPC levels above CMC displaced inhibitory CaM from RyR1 increasing its activity.
Michel et al. 2011 [14]	In vitro	Choline deficiency adversely influenced incorporation of pre-existing FA and DAG for PC synthesis, increasing TAG synthesis; CD downregulated mRNA level of choline-transporter SLC44A1.
Kenney et al. 1995 [15]	Animal study	Choline (0.18%) and myo-inositol (0.5%) consumption compared to control group, determined significant difference on liver fat percent (6.68 ± 2.23% vs. 9.22 ± 2.91%) and no difference on carcass fat percentage (24.04 ± 3.36% vs. 25.122 ± 5.83%), respectively.
Eder 1999 [16]	Animal study	Hyperlipidemic diet associated to low choline concentrations diet intake did not influence PC levels suggesting sufficient choline endogenous synthesis in geese.
Ilcol et al. 2005 [17]	Animal study	At 2 to 6 h after 0.02 mg/kg intravenous endotoxin infusion, serum-free choline concentration decreased by 14% to 49% and serum phospholipid-bound choline concentrations increased by 19% to 27%; up to 48 h after 1 mg/kg endotoxin, both serum-free and phospholipid-bound choline concentrations increased by 23% to 98% and by 18% to 53% respectively. Intravenous administration of choline (20 mg/kg) 5 min before, and up to 8 h after 1 mg/kg of endotoxin seems to reduce endotoxin-induced tissue injury, in particular on skeletal muscle.
Schenkel et al. 2014 [18]	Animal study	Palmitic Acid and Oleic Acid reduced CTL1/SLC44A expression on plasma membrane and mitochondrial membrane, modulating choline cytoplasmatic content and its bioavailability for PC synthesis.
Li et al. 2015 [19]	Animal study	Diet supplementation with 0.25% RPC increased growth, intramuscular fat and expression of genes involved in lipogenesis (CD36, ACC and FASN) in lambs.
Oster et al. 2015 [20]	Animal study	Diet supplemented with methionine, folate, choline (2230 mg/kg), B6, B12, and zinc increased fetal weight compared to those fed with standard diet at 35- and 91- dpc time points. Methionine diet reduced levels of IGFBP2 and increased IGF2 levels in skeletal muscle at 91 dpc.
Robinson et al. 2016 [21]	Animal study	Methyl deficiency diet including low choline content reduced whole-body protein synthesis (−12%, *p* = 0.01) and skeletal muscle specific protein synthesis (−60%, *p* = 0.003) compared to methyl sufficient diet.
Taylor et al. 2017 [22]	Animal study	Choline supplementation (~240 μg/g/day for 4 weeks) increased content of membrane phospholipids (PC and sphingomyelin), DAG and glycogen levels (+30% and +60%, respectively) in Pcyt2+/− mice. Conversely, choline reduced muscle TAG content of 40%, de novo FAs synthesis and lipogenesis.
Jahanian et al. 2018 [23]	Animal study	Dietary supplementation with choline (1000 mg/kg), increased protein content in leg and breast muscle. Diet with +0.42 MJ/kg ME levels plus choline improved protein content compared to ME alone in leg muscle (18.68% vs. 17.80%).
Fisher et al. 2007 [24]	Clinical study	Low choline intake provoked fatty liver or muscle damage in 33 subjects (77% of men, 80% of postmenopausal women, and 44% of premenopausal women developing organ dysfunction). During the 550-mg choline diet, also 6 men developed the same signs. All participants presented high levels of CPK. However, these negative effects reversed after choline repletion.
Mc Lean et al. 2013 [25]	Clinical study	After a 19-day of ALT an improved carnitine/choline peak (8.8% ± 6.1%) was observed in soleus muscle in athletes, compared to sea level training.

Abbreviations: Sarco-Endoplasmic Reticulum Calcium ATPase (SERCA), sarcoplasmic reticulum (SR), sphingosylphosphorylcholine (SPC), ryanodine receptor 1 (RyR1), critical micelle concentration (CMC), choline deficiency (CD), Ca^2+^ sensor calmodulin (CaM), choline transporter-like protein 1 (CTL1/SLC44A1), rumen-protected choline (RPC), cluster of differentiation 36 (CD36), acetyl-CoA carboxylase (ACC) and fatty-acid synthase (FASN), day post conception (dpc), insulin-like growth factor (IGF), insulin-like growth factor binding proteins (IGFBPs), phosphatidylcholine (PC), diacylglycerol (DAG), triglyceride (TAG), fatty acids (FAs), metabolisable energy (ME), creatine phosphokinase (CPK), and training at moderate altitude (ALT).
